# Psychosocial and treatment correlates of opiate free success in a clinical review of a naltrexone implant program

**DOI:** 10.1186/1747-597X-2-35

**Published:** 2007-11-23

**Authors:** AS Reece

**Affiliations:** 1 Southcity Family Medical Centre, University of Queensland, 39 Gladstone Rd., Highgate Hill, Brisbane, Queensland, 4101, Australia

## Abstract

**Background:**

There is on-going controversy in relation to the efficacy of naltrexone used for the treatment of heroin addiction, and the important covariates of that success. We were also interested to review our experience with two depot forms of implantable naltrexone.

**Methods:**

A retrospective review of patients' charts was undertaken, patients were recalled by telephone and by letter, and urine drug screen samples were collected. Opiate free success (OFS) was the parameter of interest. Three groups were defined. The first two were treated in the previous 12 months and comprised "implant" and "tablet" patients. A third group was "historical" comprising those treated orally in the preceding 12 months.

**Results:**

There were 102, 113 and 161 patients in each group respectively. Groups were matched for age, sex, and dose of heroin used, but not financial status or social support. The overall follow-up rate was 82%. The Kaplan Meier 12 month OFS were 82%, 58% and 52% respectively. 12 post-treatment variables were independently associated with treatment retention. In a Cox proportional hazard multivariate model social support, the number of detoxification episodes, post-treatment employment, the use of multiple implant episodes and spiritual belief were significantly related to OFS.

**Conclusion:**

Consistent with the voluminous international literature clinically useful retention rates can be achieved with naltrexone, which may be improved by implants and particularly serial implants, repeat detoxification, meticulous clinical follow-up, and social support. As depot formulations of naltrexone become increasingly available such results can guide their clinical deployment, improve treatment outcomes, and enlarge the policy options for an exciting non-addictive pharmacotherapy for opiate addiction.

## Introduction

Ever since the pathfinding studies in New York of Dole and Nyswander [1] and colleagues methadone has been the mainstay of the medical treatment of opiate addiction of patients for whom detoxification and a drug free lifestyle appears to be beyond reach. Indeed the successes of agonist replacement therapy, first with methadone and more recently with buprenorphine, have made agonist replacement therapy something of a standard of care to be sought in the treatment of other chemical addictions for which treatment as yet either does not exist or is still in its infancy. There are however increasing concerns in relation to the immunosuppressive activities of opiate [2,3] and other addictive drugs [4,5,7], and there have long been recognized significant cytostatic activities of opiates [8] and other addictive agents [9,10] which oppose cell growth and stem cell renewal which although less well known are potentially also of significant medical concern. Moreover there is increasing concern in the community about the failure of methadone to lead to a long term stable drug free lifestyle including on-going drug use, drug dealing and drug affected driving [11] together with very high rates of psychiatric symptomatology [12,13]. For practical clinical purposes however there has been little alternative for patients for whom the lifestyle and health issues or the legal constraints related to opiate addiction make medical assistance mandatory.

Naltrexone is one alternative which has existed for several decades but is rarely used. First synthesized by Matossian at Endo laboratories in 1963 [14], it was trialed clinically under sponsorship from the National Institute of Drug Abuse (NIDA) in the early 1970's and a flurry of papers were written documenting this experience. Naltrexone itself has many useful features to assist the management of opiate addiction including direct chemical blockade of opiate use for up to three days after a dose, rapid cessation of the symptoms of acute withdrawal syndrome once the initial treatment induction is accomplished, reduction of opiate craving in most patients [15-17], increasing the time to relapse to dependent use up to three days from the last administration, and in the experience of this clinic, a great reduction in the frequency with which patients think about addiction. The most well known of the early papers is that by Hollister [18] which although encouraging in terms of reporting 20% treatment retention at 7 months compared to 13% in the placebo group with many other positive adjustments in patients' lives, was felt to demonstrate a disappointingly low rate of retention in treatment. Similarly most of this early literature is similarly unenthusiastic. The major reason for this appears to be a lack of patient compliance outside of select groups such as physicians and parolees, perhaps because it does not have the addictive attraction of agonist medications, but also related to concerns of overdose and mood depression management [19,20]. Another feature of this literature is that, following the agonist literature, much of it reports treatment retention rather than non-return to dependent opiate use. Whilst this is standard practice with studies of agonist medications, non-compliance with the oral medication is not necessarily the same as dependent drug use. Studies which report "opiate free success" generally give a much more robust view of antagonist based treatment than those which focus solely on the former index [21-23].

It has been hoped that many of these compliance problems will be overcome by the recent development of depot formulations of long acting naltrexone injections and implants [24]. This was recognized very early by the National Institute of Drug Abuse which as long ago as 1976 [25] and 1981 [26] published research monographs on the subject of depot and long acting formulations of the drug. Several long acting depot injections with activity over about one month have recently been developed [27] and marketed in the USA [28] mainly for alcohol [29-31], but are also likely to find application in opiate dependent patients. More recently a longer acting device efficacious for 4–6 months [32] and used widely in Australia for opiate dependency has been developed and trialed, and is presently under consideration before the regulatory authorities in this country. Contrary to the classical view of the limited clinical role of naltrexone [33] several fascinating papers have emerged from researchers in Perth and elsewhere in relation to the use of the depot formulation in the pregnant heroin user with improved outcomes over agonist therapy [34]; the addicted anesthetist [35]; prolonged maintenance of therapeutic serum naltrexone levels above 1–2 ng/ml with sequential implants of 13–17 months [36]; potentiation of the antiviral effect of combination therapy for Hepatitis C [37] and HIV [38,39] possibly by an immunopotentiating mechanism and a dramatic truncation of overdose behaviour in high risk frequently overdosing heroin addicts [40] to the point where the earlier concerns in relation to the safety profile of naltrexone have been largely addressed [41-44]. Other centres have a similar encouraging experience with the use of such preparations in opiate dependency [24,28,45,46].

We were therefore interested to compare our early experience with a one month depot device from the USA, to the "Go Medical" implant from Perth, and to compare this experience in turn with the concurrent and historical experience with the oral formulation of naltrexone. Some of these results, and important lessons for patient management which follow from them, are presented in the following report. Serum levels of naltrexone, serious adverse events, and the experience with buprenorphine in the office management of opiate dependent patients are presented in companion papers.

## Methods

### Patient Population

The study was performed on patients presenting to a low cost private naltrexone clinic in Brisbane, Australia. The program had been in operation for over three years at the time the study was performed. Patients were divided into three groups on the basis of when they were treated and the primary treatment modality employed. Patients were not randomized. Group selection during the later period was mainly made on financial status, determined by the ability of patients' families to afford the implants. The three groups defined were:

1) "Implant": Patients having a naltrexone implant March 2000 – March 2001. Some of these implant patients had first been treated at earlier time points;

2) "Tablet": Patients treated with oral naltrexone March 2000 – March 2001;

3) "Historical": Patients treated with oral naltrexone 12 months prior to the recent period, March 1999 – March 2000.

In order to control for the effects of the passage of time it was felt important to keep the two oral naltrexone groups separate for most of the analysis as indicated, particularly in view of the changing heroin supply in Australia over the period of this study.

### Psychosocial Markers

Assignments of various psychosocial indicators was made by the treating clinician based on information disclosed during the time of patients' presentations to the clinic, and documented by retrospective review of patients' charts. Financial status, social support and spiritual belief were assigned in three groups. Patients' financial status was assigned into three strata: "pensioner" relating to those on social security alone; "average" relating to those in socioeconomic class III-V, and "white collar" relating to those in socioeconomic class I-II. The strength of social support networks were graded 1, 2 or 3 depending on the availability of mature adult responsible carers in the patients life, and the access of the patient to those carers. Sport and employment variables were assigned on a simple presence or absence basis. For the purposes of urine drug screening, the most recent result in the patient's file was the one analyzed. Spirituality was defined as religiosity and was assigned on the basis of declaration of belief in traditional religious belief (Buddhist, Hindu, Moslem or Judeo-Christian) (scored "2"), traditional belief and consistent practice (scored "3"), or nil, atheist or pagan (scored "1").

### Rapid Opiate Detox

The Rapid Opiate Detox and induction onto naltrexone was performed after the method of O'Neil which is similar to that approved by the NSW Health Department [47,48]. This involves two days opiate free preparation with clonidine and diazepam and other agents, and then the incremental administration of naloxone from 40 mcg IVI after a premedication with flunitrazepam, clonidine, ondansetron, octreotide and other drugs, and then the incremental administration of naltrexone, performed over 8–12 hours in a primary care setting.

### Naltrexone Preparations

Oral naltrexone tablets were used. These are the 50 mg "Revia" tablets from Du Pont, obtained in Australia from the Orphan Drug company. It was a condition of treatment in this clinic that all patients presenting for naltrexone treatment brought with them a responsible mature adult drug free carer who could supervise oral tablet administration on a daily basis, or assist with the implant process where that option was chosen. Implants were obtained from two sources both presently provided as a pre-loaded syringe. The American implants are a single pellet containing 1 g of naltrexone and were obtained from the Wedgewood Pharmacy, 405 Heron Dr. Suite 200, Swedesboro, New Jersey, USA, 08085-1749. The Australian implants contain 1.1 g naltrexone, and release about 0.4% of their dose daily. It was obtained from "Go Medical" in Perth, 200 Churchill Ave, Subiaco, Western Australia, Australia, 6008. It is also presented as 10 pellets in a sterile pre-loaded syringe.

### Implant Technique

Naltrexone implants were inserted subcutaneously after the method of Brewer. The implant may be inserted at any time after the commencement of naltrexone when the patient is stable and restful. In essence implant insertion involves the use of sterile surgical technique and local anaesthesia (with lignocaine and bupivicaine "Marcaine" and adrenaline) to make a 1.5 cm incision in the skin, and the development of a 10–12 cm. long subcutaneous tunnel by blunt dissection. The sites chosen may vary, but the most suitable are the skin of the lower abdomen or the lateral chest wall with an incision below the hair of the axilla. The pre-loaded syringe is then inserted into this space and the contents discharged. The skin is closed with two or three vertical mattress sutures.

### Follow-up

The period of follow-up was defined as one month before and after 1^st ^March 2001. Patients were followed up either in the normal course of their clinical review protocol, or for patients who had ceased clinic attendance, by formal recall by telephone and in writing. The date of the last naltrexone purchase was also obtained and factored into the calculation of the patient's likely clinical status. Urine samples and independent reports from their carer were used to verify patients' self-report. A carer was defined as a mature responsible drug free adult who was able to supervise the administration of all medication, and particularly to crush the naltrexone tablet daily and supervise its administration. The primary measure of satisfactory progress in these patients was "opiate free success." This was defined as a composite measure including having a urine drug screen negative for opiates by Thin layer Chromatography or having a carer report satisfactory progress. The date of final contact was taken as the latest of the last clinic visit, the last telephone contact, or the last naltrexone tablet to be purchased, as determined by pharmacy records. As reported below it was possible to follow up most patients. However where patients could not be followed up positively every patient was assigned a score based on how it was believed they had progressed, either on the report from their family, or the report from other patients in their network. Patients for whom no data was available or who were known to have relapsed to regular heroin or methadone use were presumed to have failed treatment.

### Data and Statistics

All data was obtained by retrospective review of patient charts after deliberate follow-up as described. Data was entered into Microsoft Excel spreadsheets. It was analyzed by several programs, particularly the survival and failure time analysis module of Statistica. The modes used in this module were the two sample, k-samples, and Cox proportional hazard multivariate models. Key statistics calculated were the WW statistic, the sum of scores of the differences between R1 and R2 being the number of scores below and above each score respectively, and Gehan's modification of the Wilcoxon test statistic. The statistical programs EpiInfo (CDC Atlanta, Georgia), the Mann-Kendall test for trends [49,50] including calculation of the Sens estimate for quantification of trends on the software of the University of Linkoping, Sweden [51] and the Finnish Meteorological Institute [52] were also employed. The major data output form was the Kaplan Meier Survival Curve which was generated from this data and used to illustrate rates of opiate free success. The default statistical test for significance between different Kaplan Meier rates was Gehan's Wilcoxon test. Multivariate analysis in the Survival Analysis module was by Cox's proportional hazards model in the three groups defined above. P < 0.05 was considered significant.

### Ethical Statement

This study was undertaken as part of a retrospective clinical audit of patients treated under semi-urgent conditions. Implant patients were treated under the special provisions of the Australian Special Access Scheme of the therapeutics Goods Administration in Canberra A.C.T., Australia. The study was approved by the clinic Human Research Ethics Committee, National Health and Medical Research Council registration number #000409. No procedure was performed on patients which would not have been done in the normal course of their clinical care. All patients were treated with their own informed witnessed consent. Only disidentified aggregated data is presented.

## Results

There were 102, 113 and 161 patients in each of the implant, tablet and historical groups respectively, a total of 376 patents. Various follow-up and demographic criteria are presented in the accompanying Table 1. The mean ages, mean duration of opiate use, sex distribution, and doses of heroin used were as indicated. There were no significant differences between the groups on these indicators. In the following discussion the consecutive ordered triplets refer to the implant, tablet and historical groups respectively.

**Table 1:** Sociodemographic profiles of three groups

	***IMPLANT***	***TABLETS***	***HISTORIC***
***General Features***			
**Follow-up Rate**	88.2%	72.6%	83.7%
**Age (years)**	26.1	26.2	26.7
**% Male**	68%	65%	68%
**Years IVDU**	5.5	4.5	5.2
**Dose Heroin (g)**	0.59	0.53	0.47
			
***Financial Status***			
**% Pensioners**	9%	29%	18%
**% SES III-V**	64%	59%	71%
**% SES I-II**	27%	12%	10%
			
***Social Support***			
**Weak**	25%	2%	34%
**Average**	51%	82%	58%
**Strong**	25%	16%	8%

When however the financial and social status of the three groups were compared significant differences were revealed between the various groups. These various ratios are given in the later part of Table 1. Note that the groups were not assigned randomly. Based on a numbers in the "Pensioner" category, the differences in financial means from the implant group were significant. There were more patients in the pensioner category in the tablet group (Chi Squ. = 68.48, df = 1, O.R. = 18.15, 95% C.I. = 7.85 – 43.20, P ≤ 0.001), and the historical' group (Chi Squ. = 12.54, df = 1, O.R. = 3.77, 95% C.I. = 1.66 – 8.77, P = 0.0004). The combined differences from the implant group were significant at (Chi Squ. = 37.52, df = 1, O.R. = 7.59, 95% C.I. = 3.53 – 16.83, P ≤ 0.001). The variables of social support were also distributed differently. The implant group appeared to have the largest number of patients in the "strong" category. Compared to the tablet group this difference was not significant (Chi Squ. = 2.87, df = 1, O.R. = 1.78 95% C.I. 0.87–3.68, P = 0.09), but compared to the larger historical group it was significant (Chi Squ. = 14.65, df = 1, O.R. = 3.84 95% C.I. 1.78–8.42, P = 0.001). When both groups were analyzed together the difference was significant (Chi Squ. = 11.29, df = 1, O.R. = 1.42, 95% C.I. 1.42–4.92, P = 0.0007). These baseline sociodemographic differences should be borne in mind in the consideration of all subsequent results.

The follow-up rate was 82% overall. The overall urine drug screen follow-up was 76%. The proportion of patients in each group whose urine tested positive for naltrexone was 70%, 52% and 39% respectively and 50.4% overall. The re-treatment rate of all patients attending this clinic is 1.4 (1301 treatments in 902 patients). The numbers of patients having various numbers of implants is indicated in Table 2. The re-implant rate in the present series was 24.5% (25 of 102 patients having more than one implant; and 102 patients having 141 implants).

**Table 2:** Numbers of implant procedures performed

*NO.'s IMPLANTS*	*NO. PATIENTS*
1	77
2	36
3	12
4	8
8	1
	
**TOTAL**	**141**

RE-IMPLANTATION RATE: 25/102 = 24.5%

**Figure 1:**
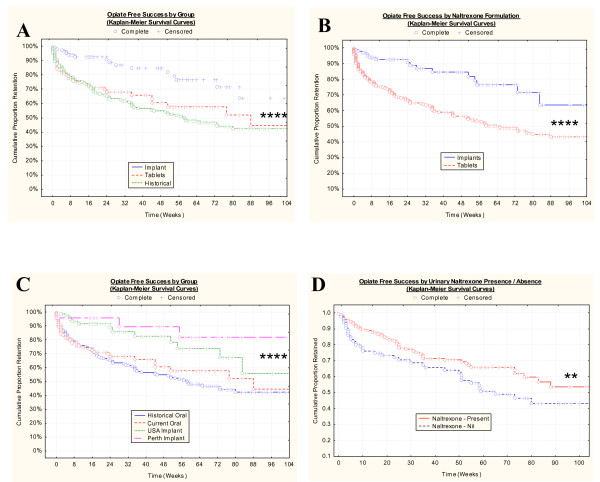
Opiate Free Success by: A.: Combined Oral Tablet and Implant Group; B: Perth, USA, Tablet and Historical Groups. C.: Presence or Absence of Urinary Naltrexone. **** – P < 0.0001; ** – P < 0.025.

Figure 1 shows the proportion of patients complying with treatment by the various treatment groups. Figure 1A shows the outcome by the three treatment groups. Figure 1B illustrates the respective outcomes by the two implant and two tablet groups conflated. Figure 1C shows the outcomes by the four treatment groups including the two different implant groups. When a statistical analysis was done of the type of implant used including no implant, there was a significant difference (Chi squared = 20.93, df = 3, P = 0.001). However when the two implants alone were compared the difference was not significant (WW = 90.00, Gehan's test statistic = 0.77, P = 0.43). Figure 1D shows the outcome by the presence or absence of naltrexone in the urine (WW = 1961.0, Gehan's test statistic = 2.4089, P = 0.1600).

Table 3 lists the urine positivity rates for various illicit drugs. The rate of obtaining a urine drug screen (UDS) for assessment in the three groups was 98%, 70% and 76% respectively. The dominant drug used was cannabis, followed by amphetamines. Amphetamine use was significantly greater in the implant group than either the tablet (Chi Squ. = 7.13, df = 1, O.R. = 2.78, 95% C.I. = 1.22–6.42, P = 0.0075) or historical (Chi Squ. = 11.14, df = 1, O.R. = 3.28, 95% C.I. 1.52–7.18, P < 0.001) groups. Amphetamine use was significantly elevated in the implant group post-treatment compared to the two oral groups combined (Chi Squ. = 13.83, df = 1, O.R. = 3.05, 95% C.I. 1.60–5.83; P = 0.0002).

**Table 3:** Urine positivity rates (%) of different illicit drugs

**Drug**	**Implant**	**Tablet**	**Historic**
Amphetamine	31	14	12
Morphine	4	5	16
THC	50	39	34
Cocaine	1	0	0
Methadone	1	1	7

Table 4 lists the improvement in work status before and after treatment for the three groups. There was a significant improvement in the implant and tablet groups, but not in the historical group. If the analysis is performed on all three groups as three groups the employment status improved from 35% prior to treatment to 54% afterwards (Chi Squ. = 27.57, df = 5, O.R. = 2.24, C.I. 1.65–3.09, P < 0.001).

**Table 4:** Work status before and after R.O.D.

	***BEFORE***	***AFTER***	WEIGHTED O.R.	95% C.I.	***SIGNIFICANCE LEVELS***
**IMPLANT**	18%	50%	4.67	2.35–9.33	<0.001
**TABLETS**	25%	48%	2.78	1.52–5.09	<0.001
**HISTORIC**	53%	60%	1.36	0.85–2.16	0.178
**OVERALL**	35%	54%	2.24	1.65–3.09	<0.001

These tests were Chi squared tests. Rows 1–3 had one degree of freedom, and row four had five degrees of freedom.

Review of spiritual status of patients after treatment revealed that 65%, 26%, and 9% scored 1, 2 and 3 respectively, with no significant differences in between group distribution.

**Figure 2:**
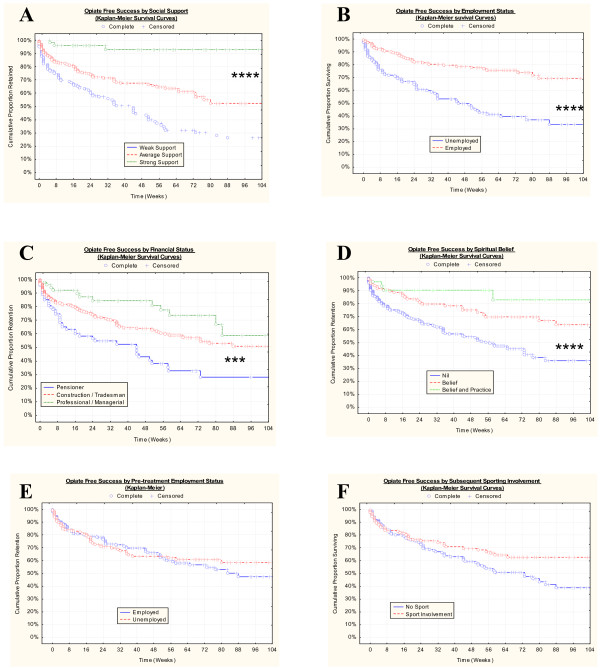
Impact of Selected Psychosocial Variables on Opiate Free Success: A.: Social support; B.: Post-treatment employment status; C.: Financial Status; Spiritual Belief; E.: Pre-treatment employment status; F.: Sporting involvement. *** P < 0.001; **** – P < 0.0001.

**Figure 3:**
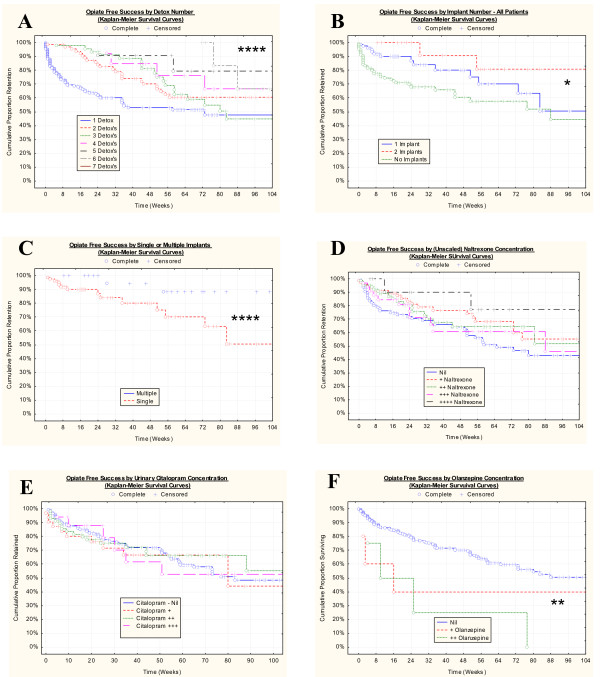
Impact of Selected Treatment Variables in Opiate Free Success: A.: No. of detox episodes; B.: No. implant episodes; C.: Single vs. multiple implant episodes; D.: Urinary naltrexone levels; E.: Urinary citalopram levels; F.: Urinary olanzepine levels. * – P < 0.05; ** – P < 0.025; **** – P < 0.0001.

**Figure 4:**
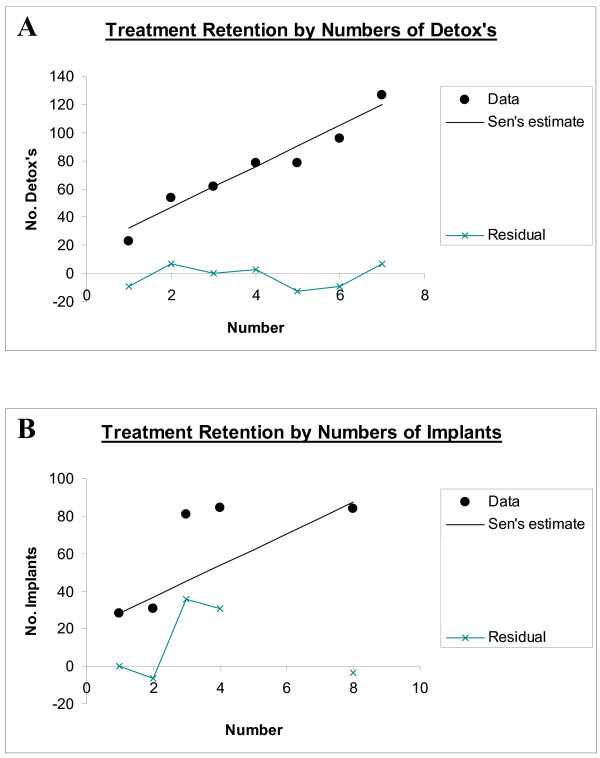
Significant Treatment Trend Analysis: A.: Detox episodes; B.: Implant episode numbers

Table 5 lists detailed outcome data by various treatment parameters. Figure 2 shows the outcome for selected psychosocial variables, and Figure 3 illustrates outcomes for selected treatment variables. Table 6 lists the bivariate significance of these various relationships. Dichotomous and multiple response variables have been analyzed in closely related but differing statistical modules within the software giving rise to the varying statistics presented for each variable. The following variables are significant on bivariate analysis: post-treatment employment, the number of detox's, social support, the use of implants rather than tablets, and the number of implants administered (all P ≤ 0.001), the treatment group in which patients were, the type of implant (as discussed above), financial status, the presence of morphine on urine drug screen, spirituality, olanzepine on urine testing, and naltrexone urine positivity were all significant (P < 0.05). Other variables identified were not significantly related to outcome. It is noteworthy that the trend for the number of implants was of borderline significance (S-score = 8, Sens slope estimate 8.44, Mann-Kendall statistic = 1.959, P = 0.0500) and for the number of detox's was highly significant (S-score = 19, Sens slope estimate = 14.56, Mann-Kendall statistic 2.853, P = 0.0043) as illustrated for both variables in Figure 4.

**Table 5:** Detailed outcome statistics for opiate free success for selected treatment variables

	**Median**	**Mean**	**Std. Dev.**	**% Completed**	**% Censored**	**Total N**
**No. Detox's**						
1	15.29	23.09	24.64	38.74	61.26	222
2	55.00	53.82	30.07	35.38	64.62	65
3	55.00	61.43	30.00	40.00	60.00	45
4	76.86	78.56	35.66	30.77	69.23	13
5	66.86	78.19	34.35	18.18	81.82	11
6	95.29	95.66	21.45	25.00	75.00	8
7	126.64	126.64	1.92	50.00	50.00	2
Total	25.00	39.04	34.45			366
						
**No. Implants**						
0	27.29	40.22	35.73	46.72	53.28	274
Single	16.29	28.32	27.57	18.18	81.82	77
Multiple	57.71	56.07	32.49	8.00	92.00	25
Total	25.00	38.84	34.57			376
						
**Type of Treatment**						
Historicals	56.86	48.83	36.94	55.28	44.72	161
Tablets	21.57	27.96	30.06	34.51	65.49	113
USA	17.14	30.59	28.68	16.67	83.33	78
Perth	51.36	49.84	34.56	12.50	87.50	24
Total	25.00	38.84	34.57			376
						
**Urinary Naltrexone**						
Absent	36.00	43.48	36.33	47.06	52.94	85
UDS +	30.14	43.75	35.95	27.54	72.46	69
UDS ++-++++	24.00	37.96	32.76	27.93	72.07	111
Total	27.29	41.24	34.75			265

Note on Table interpretation. The first three columns of this table list the days of opiate free success. "Completed" observations refers to patients whose clinical course has been determined be there time of follow-up. Censored observations refer to those patients still in treatment at the time of follow-up, and whose final outcome in treatment therefore remains to be determined at a future time point.

**Table 6:** Bivariate correlations of opiate free success. Kaplan – Meier survival curves. All patients.

Parameter	WW	Chi Squared	Gehan's – Wilcoxon Test Statistic	Degrees Freedom	P
Work After	6582.0		5.1978		0.00000
No. Detox's		39.1498		6	0.00000
Social Support		34.5710		2	0.00000
Implants or Tablets	6427.0		4.4719		0.00001
No. Implants		22.2834		2	0.00001
Group		20.4844		2	0.00004
Type of Implant		20.9302		3	0.00011
Financial Status		16.3870		2	0.00028
Morphine +UDS		18.4386		3	0.00036
Spirituality		19.5863		2	0.0006
Olanzepine +UDS		7.8996		2	0.01927
Naltrexone Simplified +UDS		6.0243		2	0.0492
					
Ecstacy +UDS		5.3881		3	0.14551
Cannabis Bivariate	1199.0		-1.3281		0.18416
Sport After	1324.0		1.0902		0.27561
Benzodiazepine +UDS		2.1997		2	0.33293
Cannabis Trivariate		1.7659		2	0.41357
Olanzepine Simple	458.00		1.9468		0.51156
Amphetamine + UDS		0.9888		2	0.60994
Heroin/Methadone	-392.0		0.4169		0.67676
Citalopram +UDS	306.00		0.3537		0.72353
Citalopram Bivariate +UDS		0.3563		2	0.83683
Work Before	-245.0		-0.1958		0.84477
Male Sex	72.000		0.0469		0.96255

Note: This table combines statistical results for dichotomous variables and also variable with multiple responses. The Chi squared and degrees of freedom columns apply to the dichotomous variables, and the WW and Gehan's test statistic columns apply to variables with multiple categories. Drug levels in urine have been "simplified" or combined into composite trivariate categories to aide statistical analysis by combining levels of ++ to ++++ as simply ++. Parameters are presented as ordered by significance of the P-value.

Clearly however the central issue relates to relative importance of these various factors. A multivariate survival analysis was performed which demonstrated that social support, the number of detox's, work after treatment, type of implant, the number of implants and spirituality continue to be significant. When a similar analysis was performed limited to implant patients alone, social support, work prior to treatment and the presence of cannabis in the urine were significantly related to opiate free success.

## Discussion

This study has been performed as part of an ethically approved clinical audit of the opioid treatment program in this clinic and makes pertinent comparisons between rates of opiate free success in patients treated with implant and orally administered naltrexone as well as a variety of psychosocial and treatment parameters. Factors which were noted on bivariate analysis to be significant were in order were employment subsequent to treatment, the number of detox episodes, the level of social support, the number of implant episodes, whether implants or tablets were used, the use of multiple implants, the type of implant (including no implant), spirituality, the financial status of the family unit, the presence of morphine in the urine, the presence of naltrexone in the urine, negatively correlated with the presence of olanzepine in the urine and the semi-quantitative presence of naltrexone in the urine (Table 6). On multivariate regression the significant variables were the strength of social support, the number of detox episodes, work after treatment, the type of implant (including none), the use of multiple implant episodes, and spiritual belief. When the multiple regression was limited to implant patients alone the only variables of significance were social support, work prior to treatment and the presence of cannabis in the urine. In this group the number of implants was of borderline significance (P = 0.06).

With the increasing availability of long acting and depot preparations of antagonists onto the pharmaceutical market some guidelines for their application would be most useful. It must be noted in considering the outcome analysis of this study that the form of the present work was a retrospective chart review. Patients were not randomized and there were notable differences between the groups at enrolment in social support and financial status which were related to having to fund the cost of the treatment including implants. Given this important limitation there are a number of indications from this data which are useful for directing clinical practice, and may be tested in well designed randomized clinical trials. Strengths of this study included the relatively high overall follow up rate of 82%, the relatively large patient numbers reviewed, the breadth of treatment and psychosocial factors examined, and the various formulations of naltrexone used.

The major point of this study is the remarkable success of sustained implant therapy. 88% of the Perth implant patients and 78% of the USA implant patients were opiate free at 12 months. Our data clearly indicates that repeated episodes of detoxification and of implant insertion are important contributors to opiate free success and to the long term engagement with patients whose long term course is recognized to be marked by periodic relapse. This notable success rate suggests a potential alternative to other management streams for refractory opiate dependence such as rehabilitation where places are typically limited, and incarceration with all its attendant costs and problems. This latter option has been extensively discussed [53,54].

We did not find a difference in the two types of implant used. Whilst this may be due to considerations of statistical power, the null hypothesis cannot be confidently excluded on the basis of the present data. It is noteworthy that the surgical procedure for implant insertion is straightforward. Since this study was completed the usual technique has changed in that rather than precipitate an acute detoxification syndrome under sedation using oral or parenteral antagonists as described here, a generally milder procedure is performed where the implant itself is used to detoxify the patient. As the serum levels achieved with the subcutaneous route are lower than those seen with alternative routes in well prepared patients, this technique is easier and much faster, and suited to ambulatory use.

We were pleased to note a 54% opiate free success rate for tablet administration which is higher than that reported from many other centres. However it should be noted that this figure includes supervised and assisted administration of the tablet by a significant other individual, opiate free success rates rather than simply retention only, and repeated episodes of detoxification and treatment initiation. Whilst this is a very different design from most naltrexone studies in the literature, it is nonetheless normal practice in both other addiction pharmacotherapies and most drug free rehabilitation programs. Careful involvement of the residual functional social network is an important buttress to pharmacological treatment, including supervision of the taking of the oral tablet, crushing the tablet and intensive medical supervision of minor side effects in the early post-treatment period, which all appear to be important to optimize results. The involvement of supervision might explain the classical rather paradoxical perception that naltrexone appears to work best for professionals and recent prisoners [55], which stereotypically represent the highest and lowest end of the social spectrum.

The literature experience with SSRI antidepressants appears mixed [56,57]. In the present project no useful effect was found. The presence of olanzepine in urine correlated negatively with opiate free success, which is not surprising given its use in psychotic and the most severe mood disorders. The elevated rate of amphetamine usage amongst the implant cohort is of concern, albeit not perhaps unexpected given the persistence of the ritual of intravenous drug use in a milieu in which opiate use is abruptly truncated. Unpublished data suggests that amphetamine use shows a steady decline after an initial surge after implant insertion (O'Neil personal communication) and our results would be consistent with this pattern. It is likely that one of the major triggers to stimulant use is the physical exhaustion which follows a an induced detoxification period but further work is required to delineate the natural history of such activity amongst implant patients, and this is understood to be an area of active investigation at this time.

The significant and dramatically increased rates of work involvement after treatment is gratifying. This is also of therapeutic importance in that it was shown to correlate with success in both univariate and multivariate regression. Privileged financial status (as defined in the Methods section) whilst having some bearing on success in a bivariate study, was shown not be significant when treatment success was controlled for social support and spirituality, which appeared to be the proximate underlying and determinative variables.

The prominence of spirituality in the statistical analysis of this study is of some interest. This area is increasingly becoming a focus of research interest in recent times [58,59]. Probably in common with most drug dependent cohorts, the major religious affiliation of these patients was "not involved/no belief". As this clinic had only four Muslim families and two Hindu in the present sample, the major religion or background of these patients was Christian which is in keeping with the major religious framework in Australia. The strong effects persisting on multivariate analysis are of interest. They may in part be related to the inclusion of a category of religious life practice and consistency in this rating in which drug use was a factor. The salience of this factor in this study is consistent with previous demonstrations of the centrality of an holistic understanding both of the psychopathology of addiction and of the road to recovery [60,61,63], the acknowledged success of 12-step programs, with classic statements on the centrality of religion to character formation and development [64] and also with the enduring holistic definition of Health itself as proposed by WHO [65]. Spiritual pursuits offer obvious mechanisms to cope with the some of the major issues of the psychological agenda for recovery including refuge and solace, peace, joy, coping with stress and guilt, values and virtue transformation, as well as an alternative source for the elusive paradise and esoteric pathway sought by many [66].

As mentioned above this paper does not discuss adverse outcomes which are addressed in a companion study. In general terms it can be said that this clinic has not encountered a mortality concern with these long acting implants in the present experience no patient has died related to causes which are attributable to the implant. A useful margin of safety however must form a pillar of successful adoption of a new treatment if it is to be widely deployed. The recent study of the Perth cohort by Hulse was noted above, and the overall mortality risk of naltrexone implant management was shown to be at least as low as that of methadone maintenance [44]. A further and very reassuring data source of interest was a recent Australia wide study which examined all coronial reports in the whole country which mentioned naltrexone implants. Of the several thousand Perth implants which have been used in Australia, only a single case was identified where a naltrexone implant was plausibly temporally implicated in the mode of death in which metamphetamine and cocaine metabolites were also present and the level of naltrexone measured in the serum suggested a problematic assay technique.

There are several limitations of the above study. Some of these relate to the design limitations of a clinical audit review. These would include the non-randomized nature of the study, the use of historical controls, and the need of patients to provide both drug free carers to supervise their treatment, and to fund the costs of their treatment themselves. One limitation mentioned above was the smaller numbers of patients with Perth implants which may have lacked statistical power to define a difference between the two implant types. There are several indications that the experience reported herein is preliminary in that this clinic lacks associated services which may be found with larger programs such as counsellors, integrated Hepatitis C care, gynecological care [67] and assisted accommodation entry, all of which would ideally be part of a demonstration or pilot program, and may receive consideration for inclusion in a formal randomized trial.

A further physiological point of some interest is the general consideration of the health effects of naltrexone, which in general may be said to be opposite to those of opiates. The effect on tissue growth and cell division are of particular interest although they would appear to have received little research attention [68]. In this regard the recent demonstration in four genome wide screens in three reports that the locus most strongly associated with coronary artery disease on the human genome encodes the two cyclin dependent kinases P16INK4A and P15INK4B is fascinating [69-71]. It is relevant to the present therapeutic study by virtue of the known potentiation of apoptosis [72-76] and impairment of cell growth by addictive drugs [77-79] which together suggest an effect to accelerate the ageing process, and by the demonstrated effect of naltrexone to reverse such effects on cell growth [80]. Furthermore evidence of an acceleration of the atherogenesis by addictive drugs has been published in recent years from Johns Hopkins hospital [81], and the NIH intramural laboratories [82], from the National Drug and Alcohol Research Centre in Sydney [83] and has been confirmed in this clinical population (unpublished observations). Indeed biochemical, immune and haematological evidence for such a progeroid effect of addiction was recently published [84].

Evidence of quantitative circulating stem cell deficits in addiction [85], together with clinical evidence of stem cell deficiencies in hair [86], bone [87] and teeth [88] together with substantial impairment of body growth [89] have recently been reported. In the study of dental pathology [88] morphine and methadone dose were shown in multivariate regression to be significant correlates of the disorder of erosive periodontitis in which both immune and stem cell components are believed to play major roles. That naltrexone might reverse such an effect [90,91], and that this may be demonstrable by assay of the classical molecules of cell growth [92]and senescence [93,94] in somatic and stem cell tissues is an intriguing possibility, well worth further investigation in view of the elevated rate of many pathologies in addiction [95] and the rapid age related accrual of hepatic, cardiac, pulmonary, renal and multi-system pathology amongst decedent IVDU's [83], not to mention the very high mortality associated with long term substance dependence [96,97]. A recent comment by the scientific leaders of addiction medicine that indeed the lead candidates involved in nicotine addiction were molecules involved in intracellular machinery is a further intriguing clue in this regard [98].

## Conclusion

In summary, this study confirms extensive literature experience that both oral and implantable naltrexone can be used with gratifying results in a primary care setting. Open access to affordable treatment appears to be important to ensure maintained success rates. The observed 12 month opiate free success rate of 54% with tablets could possibly be raised to 82% by the use of subcutaneous implants. Whilst it seemed to the clinician involved that the longer lasting Perth implants may be superior to those which are presently in more widespread use, the present study evidently lacked sufficient statistical power to show this. Ready access to repeat treatment and particularly repeat depot implantation would appear to be key components of a program seriously endeavoring to deliver high quality outcomes, and would appear to have the capacity to dramatically raise the treatment potential and patient appeal of naltrexone therapeutics. It is important not to overlook likely benefits to health of such medium term opiate antagonism including immunostimulation and recovery of cellular growth and organismal dysfunction which is likely to have accrued over a period of drug dependency. Employment after treatment, cannabis use, and spiritual development were all shown to be significant. It would seem likely that in the future implants will become the route of administration of choice for naltrexone. Subsequent to the completion of successful trials in the USA and Perth, extension of these findings in opiate dependent populations in prospective randomized multicentred trials would appear to be the logical next step for research and development in this area. Long acting injections, and depot delivered antagonists for other drugs of addiction appear to potentially hold therapeutic promise and might prove to be the way of the future.

## Competing interests

Dr. Reece reports that the naltrexone implants mentioned in this study were sold for use in patients from his clinic during the period of this study.

## Authors' contributions

ASR conceived and designed the study, perfoemed the literature review, coordintaed the follow up, drafted the manuscript, perforemed the statistical analysis, and constituted the graphical illustrations. He has read and approved the final manuscript.

## References

[B1] Dole V, Nyswander M (1965). "A Medical Treatment for diacetylmorphine (heroin) addiction. A clinical trial with methadone hydrochloride.". JAMA.

[B2] Suzuki S, Carlos MP, Chuang LF, Torres JV, Doi RH, Chuang RY (2002). Methadone induces CCR5 and promotes AIDS virus infection.". FEBS Lett.

[B3] McCarthy L, Wetzel M, Sliker JK, Eisenstein TK, Rogers TJ (2001). "Opioids, opioid receptors, and the immune response.". Drug Alcohol Depend.

[B4] Pillai R (1991). "AIDS, Drugs of Abuse and the immune system: A Complex immunotoxicological network". Arch Toxicol.

[B5] Eisenstein TK, Meissler JJ, Wilson Q, Gaughan JP, Adler MW "Anandamide and Delta(9)-tetrahydrocannabinol directly inhibit cells of the immune system via CB(2) receptors.". J Neuroimmunol.

[B6] Klein TW, Cabral G (2006). "Cannabinoid-Induced Immune Suppression and Modulation of Antigen-Presenting Cells.". J Neuroimmune Pharmacol.

[B7] Cabral G (2006). "Drugs of Abuse, Immune Modulation, and AIDS". J Neuroimmune Pharmacol.

[B8] Zagon IS, Verdamme MF, McLaughlin PJ (2002). "The biology of the opioid growth factor receptor (OGFr).". Brain Res Brain Res Rev.

[B9] Kogan NM, Schlesinger M, Priel E, Rabinowitz R, Berenshtein E, Chevion M, Mechoulam R (2007). "HU-331, a novel cannabinoid-based anticancer topoisomerase II inhibitor.". Mol Cancer Ther.

[B10] Eisch AE, Madyam CH (2004). "Adult Neurogenesis and Drug Abuse". Am J Psychiatry.

[B11] Fischer J, Jenkins N, Bloor MJ, Neale J, Berney L (2007). User involvement in treatment decisions: final report.

[B12] Dyer KR, White JM (1997). "Patterns of symptom complaints in methadone maintenance patients.". Addiction.

[B13] Callaly T, Trauer T, Munro L, Whelan G (2001). "Prevalence of psychiatric disorder in a methadone maintenance population.". Aust N Z J Psychiatry.

[B14] Schecter A (1980). "The Role of Narcotic Antagonists in the Rehabilitation of Opiate Addicts: A Review of Naltrexone.". Am J Drug Alcohol Abuse.

[B15] Sideroff SI, Charuvasta VC, Jarvik ME (1978). "Craving in Heroin Addicts Maintained on the Opiate Antagonist Naltrexone.". Am J Drug alcohol Abuse.

[B16] Judson BA, Carney TM, Goldstein A (1981). "Naltrexone Treatment of Heroin addiction: Efficacy and Safety in a Double Blind Comparison.". Drug Alcohol Depend.

[B17] Guthrie SK (1990). "Pharmacologic Interventions for the Treatment of Opioid Dependence and Withdrawal.". DICP The Annals of Pharmacotherapy.

[B18] Hollister LE, Editorial (1978). "Clinical Evaluation of Naltrexone: Treatment of Opiate Dependent Individuals. Report of the National Research Council Committee on Clinical Evaluation of Narcotic Antagonists.". Arch Gen Psychiatry.

[B19] Miotto K, McCann MJ, Basch J, Rawson RA, Ling W (1997). "Overdose, suicide attempts and death among a cohort of naltrexone-treated opioid addicts.". Drug Alcohol Depend.

[B20] Miotto K, McCann MJ, Rawson RA, Frosch D, Ling W (2002). "Naltrexone and dysphoria: fact or myth?". Am J Addict.

[B21] Gerra G, Marcato A, Caccavari R, Fontanesi B, Delsignore R, Fertonani G, Avanzini P, Rustichelli P, Passeri M (1995). "Clonidine and opiate receptor antagonists in the treatment of heroin addiction.". J Subs Abuse Treat.

[B22] Washton AM, Gold MS, Pottash AC (1984). "Naltrexone in addicted physicians and business executives. NIDA Res Monogr.

[B23] Greenstien RA, Arndt IC, McLellan AT, O'Brien CP, Evans B (1984). "Naltrexone: A Clinical perspective.". J Clin Psychiatry.

[B24] Brewer C (2007). Letter in response to "Opioid overdose deaths can occur in patients with naltrexone implants.". Med J Aust.

[B25] Capozza RC, Schmitt EE, Sendelbeck LR (1976). "Development of chronomers for narcotic antagonists.". NIDA Research monograph No 4: Narcotic Antagonists: The Search for long-acting preparations.

[B26] Olsen JL, Kincl FA (1981). "A review of parenteral sustained release naltrexone systems." PP187-193. Narcotic Antagonists 1981 "Naltrexone Pharmacotherapy and sustained release preparations.". NIDA research monograph No 28.

[B27] Comer SD, Collins ED, Kleber HD, Nuwayser ES, Kerrigan JH, Fischman MW (2002). "Depot naltrexone: long-lasting antagonism of the effects of heroin in humans.". Psychopharmacology (Berl).

[B28] Comer SD, Sullivan MA, Yu E, Rothenberg JL, Kleber HD, Kampman K, Dackis C, O'Brien CP (2006). "Injectable, sustained-release naltrexone for the treatment of opioid dependence: a randomized, placebo-controlled trial.". Arch Gen Psychiatry.

[B29] Kranzler HR, Wesson DR, Billot L, DrugAbuse Sciences Naltrexone Depot Study Group (2004). "Naltrexone depot for treatment of alcohol dependence: a multicenter, randomized, placebo-controlled clinical trial.". Alcohol Clin Exp Res.

[B30] Johnson BA (2006). "A synopsis of the pharmacological rationale, properties and therapeutic effects of depot preparations of naltrexone for treating alcohol dependence.". Expert Opin Pharmacother.

[B31] Garbutt JC, Kranzler HR, O'Malley SS, Gastfriend DR, Pettinati HM, Silverman BL, Loewy JW, Ehrich EW, Vivitrex Study Group (2005). "Efficacy and tolerability of long-acting injectable naltrexone for alcohol dependence: a randomized controlled trial.". JAMA.

[B32] Ngo HT, Arnold-Reed DE, Hansson RC, Tait RJ, Hulse GK (2007). "Blood naltrexone levels over time following naltrexone implant.". Prog Neuropsychopharmacol Biol Psychiatry.

[B33] Mark TL, Kranzler HR, Song X (2003). "Understanding US addiction physicians' low rate of naltrexone prescription.". Drug Alcohol Depend.

[B34] Hulse GK, O'Neil G, Arnold-Reed DE (2004). "Methadone maintenance vs. implantable naltrexone treatment in the pregnant heroin user.". Int J Gynaecol Obstet.

[B35] Hulse GK, O'Neil G, Hatton M, Peach MJ (2003). "Use of oral and implantable naltrexone in the management of the opioid impaired physician.". Anaesth Intensive Care.

[B36] Hulse GK, Arnold-Reed DE, O'Neil G, Chan CT, Hansson RC (2004). "Achieving long-term continuous blood naltrexone and 6-beta-naltrexol coverage following sequential naltrexone implants.". Addict Biol.

[B37] Jeffrey GP, MacQuillan G, Chua F, Galhenage S, Bull J, Young E, Hulse G, O'Neil G (2007). "Hepatitis C virus eradication in intravenous drug users maintained with subcutaneous naltrexone implants.". Hepatology.

[B38] Gekker G, Lokensgard JR, Peterson PK (2001). "Naltrexone potentiates anti-HIV-1 activity of antiretroviral drugs in CD4+ lymphocyte cultures.". Drug Alcohol Depend.

[B39] Wang X, Douglas SD, Peng JS, Metzger DS, O'Brien CP, Zhang T, Ho WZ (2006). "Naltrexone inhibits alcohol-mediated enhancement of HIV infection of T lymphocytes.". J Leukoc Biol.

[B40] Hulse GK, Tait RJ, Comer SD, Sullivan MA, Jacobs IG, Arnold-Reed D (2005). "Reducing hospital presentations for opioid overdose in patients treated with sustained release naltrexone implants.". Drug Alcohol Depend.

[B41] Gibson AE, Degenhardt LJ, Hall WD (2007). "Opioid overdose deaths can occur in patients with naltrexone implants.". Med J Aust.

[B42] Hulse GK, Tait RJ (2007). "Opioid overdose deaths can occur in patients with naltrexone implants.". Med J Aust.

[B43] Arnold-Reed DE, Hulse GK, Hansson RC, Murray SD, O'Neil G, Basso MR, Holman CD (2003). "Blood morphine levels in naltrexone-exposed compared to non-naltrexone-exposed fatal heroin overdoses.". Addict Biol.

[B44] Tait RJ, Ngo HJ, Hulse GK "Mortality in heroin users three years post implant naltrexone or methadone maintenance treatment.". J Subst Abuse Treatment.

[B45] Carreño JE, Alvarez CE, Narciso GI, Bascarán MT, Díaz M, Bobes J (2003). "Maintenance treatment with depot opioid antagonists in subcutaneous implants: an alternative in the treatment of opioid dependence.". Addict Biol.

[B46] Foster J, Brewer C, Steele T (2003). "Naltrexone implants can completely prevent early (1-month) relapse after opiate detoxification: a pilot study of two cohorts totalling 101 patients with a note on naltrexone blood levels.". Addict Biol.

[B47] Bell J, Kimber J, Lintzeris N (2001). "Guidelines for Rapid Detoxification from Opioids.". Drug Programs Bureau, NSW Health Department.

[B48] Bell JR, Young MR, Masterman SC, Morris A, Mattick RP, Bammer G (1999). "A pilot study of naltrexone accelerated detoxification in opioid dependence.". Med J Aust.

[B49] Beale EM, Kendall MG, Mann DW (1967). "The discarding of variables in multivariate analysis.". Biometrika.

[B50] Libiseller C, Grimvall A (2002). "Performance of partial mann kendall tests for trend detection in the presence of covariates.". Environmetrics.

[B51] http://www.mai.liu.se/~cllib/welcome/PMKtest.html.

[B52] http://www.emep.int/assessment/MAKESENS_1_0.xls.

[B53] Caplan AL (2006). "Ethical issues surrounding forced, mandated, or coerced treatment.". J Subst Abuse Treatment.

[B54] Marlowe DB (2006). "Depot naltrexone in lieu of incarceration: A behavioral analysis of coerced treatment for addicted offenders.". J Subst Abuse Treatment.

[B55] O'Brien CP, Cornish JW (2006). "Naltrexone for probationers and parolees.". J Subst Abuse Treatment.

[B56] Landabaso MA, Iraurgi I, Jiménez-Lerma JM, Sanz J, Fernádez de Corres B, Araluce K, Calle R, Gutiérrez-Fraile M (1998). "A randomized trial of adding fluoxetine to a naltrexone treatment programme for heroin addicts.". Addiction.

[B57] Krupitsky EM, Zvartau EE, Masalov DV, Tsoy MV, Burakov AM, Egorova VY, Didenko TY, Romanova TN, Ivanova EB, Bespalov AY, Verbitskaya EV, Neznanov NG, Grinenko AY, O'Brien CP, Woody GE (2006). "Naltrexone with or without fluoxetine for preventing relapse to heroin addiction in St. Petersburg, Russia.". J Subst Abuse Treat.

[B58] Koenig HG, McCullough ME, Larson DB (2001). "Alcohol and Drug Use.". "Handbook of Religion and Health".

[B59] Keonig HG (2007). "Religion, spirituality and medicine in Australia: research and clinical practice.". Med J Aust.

[B60] Pargament KI, Maton KI, Hess RR (1991). "Religion and Prevention in Mental Health: Research Vision and Action.".

[B61] Kus RJ (1995). "Spirituality and Chemical Dependency.".

[B62] Larson DB, Swyers JP, McCullough ME (1998). "Scientific Research on Spirituality and Health: A Consensus Report.". National Institutes of Healthcare Research.

[B63] Larson DB, Matthews DA (1995). "The Faith Factor: An Annotated Bibliography of Clinical Research on Spiritual subjects.". Enhancing Life Satisfaction" National Institutes of Healthcare Research".

[B64] (1930). Sir Winston Spencer-Churchill, "My Early Life,".

[B65] Quoted by Gordon D (1976). "What is Health?". "Health Sickness and Society".

[B66] Hall W, Babor TF (2000). "Cannabis Use and Public Health: Assessing the Burden.". Editorial: Addiction.

[B67] Reece AS (2007). "Lifetime prevalence of cervical neoplasia in addicted and medical patients.". Aust NZ J Obstet Gynaecol.

[B68] Chalmers J, Ritter A, Faes C (2007). "Opioid Pharmacotherapy maintenance in Australia – A background issue paper.".

[B69] Samani NJ, Erdmann J, Hall AS, Hengstenberg C, Mangino M, Mayer B, Dixon RJ, Meitinger T, Braund P, Wichmann HE, Barrett JH, König IR, Stevens SE, Szymczak S, Tregouet DA, Iles MM, Pahlke F, Pollard H, Lieb W, Cambien F, Fischer M, Ouwehand W, Blankenberg S, Balmforth AJ, Baessler A, Ball SG, Strom TM, Braenne I, Gieger C, Deloukas P, Tobin MD, Ziegler A, Thompson JR, Schunkert H WTCCC and the Cardiogenics Consortium "Genomewide association analysis of coronary artery disease.". N Engl J Med.

[B70] McPherson R, Pertsemlidis A, Kavaslar N, Stewart A, Roberts R, Cox DR, Hinds DA, Pennacchio LA, Tybjaerg-Hansen A, Folsom AR, Boerwinkle E, Hobbs HH, Cohen JC "A common allele on chromosome 9 associated with coronary heart disease.". Science.

[B71] Helgadottir A, Thorleifsson G, Manolescu A, Gretarsdottir S, Blondal T, Jonasdottir A, Jonasdottir A, Sigurdsson A, Baker A, Palsson A, Masson G, Gudbjartsson DF, Magnusson KP, Andersen K, Levey AI, Backman VM, Matthiasdottir S, Jonsdottir T, Palsson S, Einarsdottir H, Gunnarsdottir S, Gylfason A, Vaccarino V, Hooper WC, Reilly MP, Granger CB, Austin H, Rader DJ, Shah SH, Quyyumi AA, Gulcher JR, Thorgeirsson G, Thorsteinsdottir U, Kong A, Stefansson K "A common variant on chromosome 9p21 affects the risk of myocardial infarction.". Science.

[B72] Mao J, Sung B, Ji R-R, Lim G (2002). "Neuronal apoptosis associated with morphine tolerance for an opioid-induced neurotoxic mechanism.". J Neurosci.

[B73] Maccarrone M, Lorenzon T, Bari M, Melino G, Finazzi-Agro A (2000). "Anandamide induces apoptosis in human cells via vanilloid receptors. Evidence for a protective role of cannabinoid receptors.". J Biol Chem.

[B74] Bari M, Battista N, Fezza F, Finazzi-Agro A, Maccarrone M (2005). "Lipid Rafts control signalling of type-1 cannabinoid receptors in neuronal cells. Implications for anandamide induced apoptosis.". J Biol Chem.

[B75] Krasnova IN, Ladenheim B, Cadet JL (2005). "Amphetamine induces apoptosis of medium spiny striatal projection neurons via the mitochondria-dependent pathway.". FASEB J.

[B76] Li G, Xiao Y, Zhang L (2005). "Cocaine induces apoptosis in fetal rat myocardial cells through the p38 mitogen-activated protein kinase and mitochondrial/cytochrome c pathways.". J Pharmacol Exp Ther.

[B77] Eisch AJ, Mandyam CD (2004). "Drug Dependence and Addiction II: Adult Neurogenesis and Drug Abuse.". Am J Psychiatry.

[B78] Yamaguchi M, Suzuki T, Seki T, Namba T, Juan R, Arai H, Hori T, Asada T (2004). "Repetitive cocaine administration decreases neurogenesis in the adult rat hippocampus.". Ann NY Acad Sci.

[B79] Zagon IS, Verderame MF, McLaughlin PJ (2002). "The biology of the opioid growth factor receptor (OGFr).". Brain Res Brain Res Rev.

[B80] Zagon IS, Verdamme MF, McLaughlin PJ (2002). "The biology of the opioid growth factor receptor (OGFr).". Brain Res Brain Res Rev.

[B81] Lai S, Lima JA, Lai H, Vlahov D, Celentano D, Tong W, Bartlett JG, Margolick J, Fishman EK "Human immunodeficiency virus 1 infection, cocaine, and coronary calcification.". Arch Intern Med.

[B82] Wang M, Zhang J, Spinetti G, Jiang LQ, Monticone R, Zhao D, Cheng L, Krawczyk M, Talan M, Pintus G, Lakatta EG (2005). "Angiotensin II activates matrix metalloproteinase type II and mimics age-associated carotid arterial remodeling in young rats.". Am J Pathol.

[B83] Darke S, Key S, Duflou J (2006). "Systemic disease among cases of fatal opioid toxicity.". Addiction.

[B84] Reece AS "Does addiction accelerate ageing? Immune and laboratory biomarkers of ageing in drug addiction.". Immunity and Ageing.

[B85] Reece AS, Davidson P (2007). "Deficit of circulating stem – progenitor cells in opiate addiction: A pilot study.". Substance Abuse Prevention Policy and Treatment.

[B86] Reece AS (2007). "Hair graying in substance addiction.". Arch Dermatol.

[B87] Kim TW, Alford DP, Malabanan A, Holick MF, Samet JH (2006). "Low bone density in patients receiving methadone maintenance treatment.". Drug Alcohol Depend.

[B88] Reece AS (2007). "Dentition of addiction in Queensland: poor dental status and major contributing drugs.". Aust Dent J.

[B89] Swanson JM, Elliott GR, Greenhill LL, Wigal T, Arnold LE, Vitello B, Hechtman L, Epstein JN, Pelham WE, Abikoff HB, Newcom JH, Molina BS, Hinshaw SP, Wells KC, Hoza B, Jensen PS, Gibbons RD, Hur K, Stehli A, Davies M, March JS, Conners CK, Caron M, Volkow ND (2007). "Effects of stimulant medication on growth rates across 3 years in the MTA follow-up.". J Am Acad Child Adolesc Psychiatry.

[B90] Zagon IS, Verdamme MF, McLaughlin PJ (2002). "The biology of the opioid growth factor receptor (OGFr).". Brain Res Brain Res Rev.

[B91] Janzen V, Scadden DT (2006). "Stem Cells: Good bad and reformable". Nature.

[B92] Collado M, Serrano M (2005). "The senescent side of tumour suppression.". Cell Cycle.

[B93] Beausejour CM, Campisi J (1996). "Ageing: balancing regeneration and cancer.". Nature.

[B94] Janzen V, Forkert R, Fleming HE, Saito Y, Waring MT, Dombkowski DM, Cheng T, DePinho RA, Sharpless NE, Scadden DT "Stem-cell ageing modified by the cyclin-dependent kinase inhibitor p16INK4a.". Nature.

[B95] Gottschalk LA, McGuire FL, Heiser JF, Dinovo EC, Birch H, Eds (1979). "Drug Abuse Deaths in Nine Cities: A survey report" NIDA Research monograph No. 29. US Department of Health and Human Services.

[B96] Darke S, Degenhardt L, Mattick R (2007). "Mortality amongst illicit drug users: epidemiology, causes and intervention.".

[B97] Gronbladh L, Gunne L (1989). "Methadone-assisted rehabilitation of Swedish heroin addicts.". Drug Alcohol Depend.

[B98] Li T, Volkow ND, Baler RD, Egli M (2007). "The biological basis of nicotine and alcohol co-addiction.". Biol Psychiatry.

